# Pharmacogenomics in Solid Tumors: A Comprehensive Review of Genetic Variability and Its Clinical Implications

**DOI:** 10.3390/cancers17060913

**Published:** 2025-03-07

**Authors:** Rodrigo Sánchez-Bayona, Camila Catalán, Maria Angeles Cobos, Milana Bergamino

**Affiliations:** 1 Medical Oncology, Hospital Universitario 12 de Octubre, 28041 Madrid, Spain; macobos.imas12@h12o.es; 2 Medical Oncology, Universidad Finis Terrae, Santiago 7501014, Chile; catalancamila@gmail.com; 3 Medical Oncology Department, Hospital Clinic of Barcelona, 08036 Barcelona, Spain; bergamino@clinic.cat

**Keywords:** pharmacogenomics, personalized medicine, oncology

## Abstract

Pharmacogenomics, the study of how genetic variations influence drug response, has become integral to cancer treatment as personalized medicine evolves. This review aims to explore key pharmacogenomic biomarkers relevant to cancer therapy and their clinical implications, providing an updated and comprehensive perspective on how genetic variations impact drug metabolism, efficacy, and toxicity in oncology. Genetic heterogeneity among oncology patients significantly impacts drug efficacy and toxicity, emphasizing the importance of incorporating pharmacogenomic testing into clinical practice. Genes such as *CYP2D6*, *DPYD*, *UGT1A1*, *TPMT*, *EGFR*, KRAS, and *BRCA1/2* play pivotal roles in influencing the metabolism, efficacy, and adverse effects of various chemotherapeutic agents, targeted therapies, and immunotherapies. For example, *CYP2D6* polymorphisms affect tamoxifen metabolism in breast cancer, while *DPYD* variants can result in severe toxicities in patients receiving fluoropyrimidines. Mutations in *EGFR* and *KRAS* have significant implications for the use of targeted therapies in lung and colorectal cancers, respectively. Additionally, *BRCA1/2* mutations predict the efficacy of PARP inhibitors in breast and ovarian cancer. Ongoing research in polygenic risk scores, liquid biopsies, gene–drug interaction networks, and immunogenomics promises to further refine pharmacogenomic applications, improving patient outcomes and reducing treatment-related adverse events. This review also discusses the challenges and future directions in pharmacogenomics, including the integration of computational models and CRISPR-based gene editing to better understand gene–drug interactions and resistance mechanisms. The clinical implementation of pharmacogenomics has the potential to optimize cancer treatment by tailoring therapies to an individual’s genetic profile, ultimately enhancing therapeutic efficacy and minimizing toxicity.

## Simple Summary

Pharmacogenomics is a rapidly evolving field that is crucial in optimizing cancer treatments by tailoring therapies to individual patients’ genetic profiles. This review highlights the impact of key genes, such as *CYP2D6*, *DPYD*, and *UGT1A1*, which influence the metabolism of essential cancer drugs like tamoxifen, fluoropyrimidines, and irinotecan. Variations in these genes can affect drug efficacy or increase the risk of side effects, underscoring the need for genetic testing to guide treatment decisions. Despite its proven benefits, pharmacogenomic testing is not yet widely used. Ongoing research aims to improve the accessibility and cost effectiveness of these tests, especially in regions where testing is limited. In the future, combining pharmacogenomic data with emerging technologies, such as liquid biopsies and gene-editing tools, will improve personalized cancer therapies, leading to better treatment outcomes and fewer side effects for patients.

## 1. Introduction

**Figure 1:**
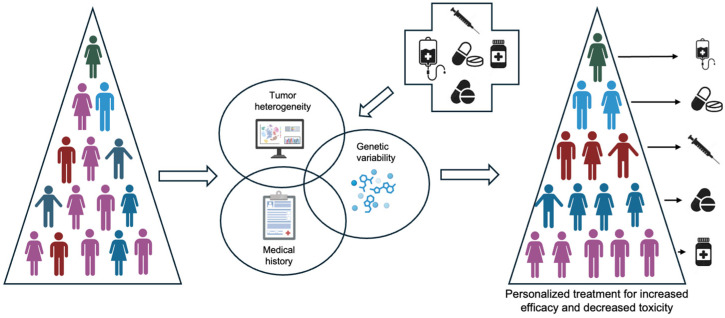
The aims of personalized medicine include the understanding of genetic variability, tumor heterogeneity, and medical context (health records, medical imaging, staging).

Pharmacogenomics has emerged as a vital field in oncology, with its potential to tailor cancer treatments based on each patient’s genetic makeup. As precision medicine evolves, understanding the interplay between genetic variations and drug response plays a critical role in optimizing cancer care (Figure 1). These variations impact not only drug metabolism but also treatment efficacy and the risk of adverse effects [1].

Adverse effects related to oncology treatment are a common subject; studies indicate that more than 50% of patients with advanced disease had one or more moderate to severe adverse events related to chemotherapy that may lead to a worsening quality of life, discontinuing treatment, increasing the risk of drug interactions due to polypharmacy, and resulting in hospitalization or death [2]. Because of the complexity of oncology patients, some studies also describe the enormous economic burden for the health system, with an average annual percentage change in these hospitalizations of 8.1%, compared to 0.5% for general hospitalization [3].

Given these challenges, numerous pharmacogenomic biomarkers have been identified. These biomarkers can predict how patients will respond to chemotherapy, targeted therapies, and immunotherapies, paving the way for more personalized cancer treatments.

Key genes, such as Cytochrome P450 enzymes, DPYD, and UGT1A1, have been identified as critical biomarkers that affect the metabolism of widely used chemotherapeutic agents like tamoxifen, cyclophosphamide, tyrosine kinase inhibitors, fluoropyrimidines, and irinotecan, leading to variability in patient outcomes. These metabolic differences can lead to significant variability in patient outcomes, underscoring the importance of genetic profiling before treatment decisions are made.

Several pharmacogenomic biomarkers have been identified that predict response to chemotherapy, targeted therapies, and immunotherapies, enabling more personalized and effective approaches for cancer treatment. In addition to guiding drug selection and dosing, pharmacogenomic insights also help identify patients at higher risk of developing severe toxicities. As the field advances, the integration of pharmacogenomic data into routine clinical practice offers the potential to transform oncology care.

This review discusses key pharmacogenomic biomarkers relevant to cancer therapy, their clinical implications, and the latest research in the field. It also explores genetic polymorphisms associated with drug metabolism, drug transporters, and molecular targets, while highlighting future directions in pharmacogenomic research aimed at enhancing treatment outcomes and reducing toxicity. Genetic heterogeneity among oncology patients significantly impacts drug efficacy and toxicity, emphasizing the importance of incorporating pharmacogenomic testing into clinical practice.

## 2. Key Pharmacogenomic Genes with Clinical Implications

### 2.1. Cytochrome P450 Enzymes

Cytochrome P450 enzymes (CYPs) are a superfamily of enzymes that play a critical role in the metabolism of a wide range of chemotherapeutic agents and other drugs used in cancer treatment. The activity of these enzymes is governed by genetic polymorphisms, leading to significant interindividual variability in drug pharmacokinetics among individuals. Understanding these genetic variations is crucial for optimizing treatment regimens, improving therapeutic outcomes, and reducing adverse effects. This section explores the clinical relevance of key cytochrome P450 enzymes: CYP2D6, CYP2C19, and CYP3A4, and their clinical relevance in oncology [4,5,6] (Table 1).

#### 2.1.1. CYP2D6 and Tamoxifen Metabolism

CYP2D6 is perhaps the most extensively studied CYP enzyme in the context of pharmacogenomics and cancer. CYP2D6 has numerous endogenous substrates including tamoxifen, a selective estrogen receptor modulator (SERM) commonly used to treat estrogen receptor-positive (ER+) breast cancer [7,8,9,10,11]. Tamoxifen is a selective estrogen receptor modulator (SERM) commonly used in the treatment of estrogen receptor-positive (ER+) breast cancer. It functions as a prodrug, requiring metabolic activation primarily by cytochrome P450 enzymes, particularly CYP2D6, to convert it into its most potent active metabolite, endoxifen. This metabolite is essential for exerting the drug’s anti-estrogenic effects on breast tissue, inhibiting cancer-cell proliferation and reducing the risk of recurrence. Variability in CYP2D6 enzyme activity can influence the effectiveness of tamoxifen therapy [12].

CYP2D6 exhibits significant genetic variability, with over 100 known polymorphisms that influence enzyme activity, resulting in phenotypes ranging from poor metabolizers (PM) to ultra-rapid metabolizers (UM) [13]. Poor metabolizers have reduced or absent enzyme activity to no CYP2D6 activity, leading to lower levels of active endoxifen and potentially suboptimal treatment outcomes. Studies have demonstrated that breast cancer patients with poor metabolizer phenotypes face a higher risk of disease recurrence when treated with standard doses of tamoxifen [14,15,16]. Conversely, ultra-rapid metabolizers have increased CYP2D6 activity, producing higher endoxifen concentrations and potentially better treatment responses [13,17].

Pharmacogenomic testing for *CYP2D6* variants has been suggested to guide tamoxifen therapy, especially for poor metabolizers who may benefit from alternative treatments or dose adjustments [14,15]. While some clinical guidelines recommend *CYP2D6* genotyping, its routine use remains controversial due to inconsistent findings across studies [18]. However, there is a growing consensus on its potential utility, particularly in patients undergoing long-term tamoxifen therapy [17,19].

#### 2.1.2. CYP2C19 and Cyclophosphamide Activation

CYP2C19 is another vital enzyme from the cytochrome P450 family, playing a key role in the metabolism of several chemotherapeutic agents, including cyclophosphamide [20]. Cyclophosphamide, a prodrug commonly used to treat lymphoma, breast cancer, and ovarian cancer, requires bioactivation by CYP2C19 and other enzymes, such as CYP2B6, to produce active metabolites that exert cytotoxic effects on cancer cells [17,21].

Genetic polymorphisms in the *CYP2C19* gene result in distinct metabolizer phenotypes. PMs exhibit reduced enzymatic activity, which may impair cyclophosphamide activation and lower the production of active metabolites, potentially diminishing therapeutic efficacy. In contrast, UMs display enhanced enzymatic activity, potentially improving drug activation and efficacy, but with an increased risk of toxicity, such as myelosuppression [20]. The clinical utility of *CYP2C19* genotyping to guide cyclophosphamide dosing remains under investigation. Preliminary studies suggest that patients with reduced CYP2C19 activity may benefit from dose adjustments or alternative therapies to optimize therapeutic outcomes [17,20].

#### 2.1.3. CYP3A4 and Tyrosine Kinase Inhibitors (TKIs)

CYP3A4 is a critical enzyme in the cytochrome P450 family, responsible for metabolizing about 50% of all clinically used drugs, including several TKIs such as imatinib, erlotinib, and gefitinib, used to treat chronic myeloid leukemia (CML) and non-small cell lung cancer (NSCLC) [20]. The activity of *CYP3A4* plays a significant role in the pharmacokinetics of these drugs, influencing both their efficacy and toxicity [22,23].

While CYP3A4 is less polymorphic than other enzymes, genetic variations or environmental factors, like the concurrent use of inhibitors or inducers, can still impact drug levels. Increased *CYP3A4* activity may lead to faster clearance of TKIs, resulting in subtherapeutic drug concentrations and reduced therapeutic. Conversely, decreased CYP3A4 activity can result in elevated drug levels increasing the risk of adverse events, including hepatotoxicity, diarrhea, and skin rash [24].

Given the variability in CYP3A4 activity, careful monitoring is essential for patients receiving TKIs. Adjusting drug doses or avoiding drug interactions can improve optimal drug exposure, and reduced adverse effects. While routine genetic testing for CYP3A4 polymorphisms is not yet standard practice, understanding an individual’s metabolic capacity is crucial for personalizing treatment regimens and enhancing patient-centered care in pharmacogenomics [24,25].

### 2.2. DPYD (Dihydropyrimidine Dehydrogenase)

Dihydropyrimidine dehydrogenase (*DPYD*) is a key enzyme in the metabolism of fluoropyrimidines, a class of chemotherapeutic agents that includes 5-fluorouracil (5-FU) and its oral prodrug, capecitabine. Fluoropyrimidines are widely used to treat of various cancers, including colorectal, breast, gastric, and head and neck cancers. The DPYD enzyme is responsible for the catabolism of approximately 80% of administered 5-FU into inactive metabolites.

Genetic variations in the *DPYD* gene can result in partial or complete enzyme deficiency, which significantly affects the metabolism of 5-FU and capecitabine. This can lead to an increased risk of severe, and sometimes fatal, toxicities in patients [26].

This section will explore the importance of DPYD in the pharmacogenomics of fluoropyrimidine-based chemotherapy, highlighting the clinical implications of DPYD polymorphisms, the current status of DPYD testing, and ongoing research into personalized dosing strategies [27,28].

#### 2.2.1. DPYD Function and the Importance of Metabolism in Fluoropyrimidines

5-FU inhibits thymidylate synthase, a key enzyme in DNA synthesis and repair, thereby preventing tumor cell proliferation. However, for this drug to exert its therapeutic effects, only a small fraction of 5-FU must be converted into active metabolites, such as fluorodeoxyuridine monophosphate (FdUMP), responsible for its cytotoxicity. Most 5-FU (around 80%) is rapidly metabolized and inactivated by the DPYD enzyme [26]. In patients with reduced or absent DPYD activity, unmetabolized 5-FU accumulates, leading to increased exposure and a higher risk of severe toxicities, including myelosuppression (neutropenia, thrombocytopenia), gastrointestinal toxicity (diarrhea, mucositis, nausea), hand–foot syndrome, cardiotoxicity, and neurotoxicity [29,30].

#### 2.2.2. DPYD Polymorphisms and Their Clinical Implications

The DPYD gene is highly polymorphic, with certain variants causing partial or complete deficiency in enzyme activity. Several well-characterized polymorphisms have been associated with significant reductions in DPYD function, each conferring varying levels of risk for fluoropyrimidine-induced toxicity (Table 2). Key *DPYD* variants include DPYD 2A (c.1905+1G>A), the most clinically relevant and well-studied mutation, which results from exon 14 skipping during mRNA processing, leading to a non-functional enzyme and significantly increasing the risk of severe toxicity in patients treated with standard fluoropyrimidine doses [25,27]. The c.2846A>T (DPYD 13) variant affects the enzyme’s substrate binding site and is associated with moderate to severe toxicity in patients receiving 5-FU or capecitabine [28] The c.1679T>G (DPYD 9B3) polymorphism causes an amino acid substitution (p.I560S) and is associated with intermediate reductions in DPYD activity, increasing the risk of toxicity [29]. Finally, the c.1236G>A (HapB3) variant, although not directly affecting DPYD activity, is in linkage disequilibrium with functional variants and is associated with increased toxicity in some populations [30].

DPYD polymorphisms significantly increase the risk of severe, potentially life-threatening toxicities in patients treated with standard fluoropyrimidine doses, especially for carriers of DPYD 2A. These toxicities can occur rapidly, requiring immediate medical intervention, with potential irreversible damage or death if undetected [27].

Due to the substantial risk, several health organizations, including the European Society for Medical Oncology (ESMO) and the Clinical Pharmacogenetics Implementation Consortium (CPIC), recommend pre-treatment genetic testing for DPYD polymorphisms to identify at-risk patients, although the adoption of DPYD testing is not universal, particularly in regions with limited testing infrastructure [31].

The management of patients with DPYD polymorphisms focuses on personalized dosing strategies. For those with partial DPYD deficiency (i.e., heterozygous carriers of DPYD 2A), dose reductions of 50% or more are recommended, with close monitoring. For patients with complete DPYD deficiency (i.e., homozygous carriers of DPYD 2A), alternative chemotherapy regimens that do not involve 5-FU or capecitabine should be considered to avoid life-threatening toxicities. Therapeutic drug monitoring (TDM) and real-time 5-FU plasma measurement can further help optimize treatment while minimizing toxicity risks [26,28,30,31].

### 2.3. Thiopurine Methyl Transferase (TPMT)

TPMT is a key enzyme involved in the metabolism of thiopurine drugs, such as azathioprine, 6-mercaptopurine (6-MP), and thioguanine. These drugs are widely used for the treatment of hematological malignancies, autoimmune disorders, and in transplant medicine. TPMT plays a critical role by catalyzing the S-methylation of thiopurines, which reduces their conversion to cytotoxic metabolites. Variability in TPMT enzyme activity, driven by genetic polymorphisms, can significantly influence both the efficacy and toxicity of thiopurine therapy [32,33] (Table 3).

#### 2.3.1. TPMT Genetic Variability and Clinical Implications

The most well-characterized *TPMT* polymorphisms include *TPMT2*, *TPMT3A*, and TPMT*3C. These genetic variants result in reduced TPMT enzymatic activity, leading to the accumulation of toxic thioguanine nucleotides (TGNs) in patients receiving standard thiopurine doses [32,34]. Individuals can be classified as normal metabolizers (wild type), intermediate metabolizers (heterozygous for low-function variants), or poor metabolizers (homozygous for low-function variants) [35]. Poor metabolizers are at a high risk of developing severe myelosuppression, a life-threatening side effect characterized by the suppression of bone marrow activity [36]. Intermediate metabolizers are also susceptible to thiopurine toxicity but to a lesser extent than poor metabolizers [20].

#### 2.3.2. TPMT Testing in Clinical Practice

To mitigate the risk of severe toxicity, pre-treatment TPMT genotyping or phenotyping is recommended in clinical guidelines before initiating thiopurine therapy [33,34,35]. Dose reductions of up to 90% are suggested for patients with two non-functional TPMT alleles (poor metabolizers), while intermediate metabolizers typically require a moderate dose reduction and careful monitoring [35]. In the absence of testing, patients often experience trial-and-error dose adjustments, increasing the risk of adverse outcomes.

#### 2.3.3. Beyond Thiopurines: TPMT and Other Therapeutics

While *TPMT* polymorphisms are predominantly associated with thiopurine drugs, studies suggest that they may also influence the metabolism and toxicity profiles of other therapeutic agents, including certain immunosuppressants and anticancer drugs [36]. As precision medicine becomes more integrated into clinical practice, *TPMT* testing serves as a model for the broader application of pharmacogenomics to optimize drug dosing and improve patient outcomes.

### 2.4. UGT1A1 (Uridine Diphosphate Glucuronosyltransferase 1A1)

Uridine Diphosphate Glucuronosyltransferase 1A1 (UGT1A1) is a crucial glucuronidation enzyme essential for detoxifying and eliminating various endogenous and exogenous compounds, including chemotherapeutic agents. In oncology, the UGT1A1 gene plays a vital role in the metabolism of irinotecan, a topoisomerase I inhibitor chemotherapy agent commonly used to treat metastatic colorectal cancer, small-cell lung cancer, and other malignancies. Polymorphisms in the UGT1A1 gene can significantly influence drug metabolism, affecting both the drug’s efficacy and toxicity [37]. This section explores the pharmacogenomics of UGT1A1, with a particular emphasis on its clinical relevance in irinotecan-based cancer therapies (Table 4).

#### 2.4.1. UGT1A1 and Irinotecan Metabolism

Irinotecan is a prodrug that is enzymatically converted by carboxylesterases into its active form, SN-38, which inhibits topoisomerase I, causing DNA damage in cancer cells. However, SN-38 is also toxic to normal cells, and its accumulation can lead to severe side effects such as neutropenia (a reduction in neutrophils, increasing infection risk), diarrhea, and mucositis. SN-38 is inactivated through glucuronidation by UGT1A1 to reduce toxicity, converting it into the inactive metabolite (SN-38G) that is excreted from the body [15,16,17,37,38,39].

The UGT1A128 polymorphism is the most clinically significant variant of the UGT1A1 gene. This polymorphism involves an additional TA repeat in the TATA box promoter region (7 repeats instead of the usual 6), reducing transcriptional activity and lowering enzyme expression. Patients who are homozygous for UGT1A128 (7/7 genotype) have significantly reduced UGT1A1 activity, resulting in decreased clearance of SN-38 and increased drug exposure [15,16,37,38]. Heterozygous carriers (6/7 genotype) exhibit intermediate UGT1A1 activity, leading to a modest reduction in SN-38 glucuronidation, placing them at some risk for toxicity, though less than homozygous carriers [39]. Individuals with the homozygous wild-type genotype (6/6) have regular UGT1A1 activity and are less likely to experience irinotecan-related toxicities at standard doses [40].

#### 2.4.2. Clinical Implications of *UGT1A1* Polymorphisms in Irinotecan Therapy

One of the most severe and dose-limiting toxicities of irinotecan therapy is neutropenia. Patients with reduced UGT1A1 activity, particularly those homozygous for *UGT1A128* (7/7 genotype), are at a significantly higher risk of developing severe (grade 3 or 4) neutropenia compared to those with the 6/6 or 6/7 genotypes [37,38,41]. Severe gastrointestinal toxicity, particularly diarrhea, which can be life-threatening, is also due to SN-38 accumulation given by impaired UGT1A1 activity [39]

In clinical practice, patients identified with the UGT1A128 polymorphism, especially those with the 7/7 genotype, are typically started on reduced doses of irinotecan of 30–50%, along with careful monitoring of blood counts and gastrointestinal symptoms during treatment to prevent severe toxicities [40]. Heterozygous carriers (6/7) may require less intense dose adjustments based on their clinical tolerance and overall health status [42].

Pre-treatment *UGT1A1* genotyping is recommended by several professional guidelines, including those from the Clinical Pharmacogenetics Implementation Consortium (CPIC) and the European Medicines Agency (EMA), particularly for patients undergoing irinotecan therapy at higher doses (≥250 mg/m^2^) [39]. Genotyping helps identify patients at increased risk of toxicity, allowing for proactive dose adjustments or the consideration of alternative therapies [43]. Studies have reported that routine *UGT1A1* testing is cost effective, reducing the incidence of severe neutropenia and hospitalization costs, especially in patients receiving high-dose irinotecan or those undergoing dose escalation [39].

In addition to genotyping, therapeutic drug monitoring (TDM) of SN-38 levels can be utilized in specific clinical settings for real-time, personalized dose adjustments based on drug exposure. This approach is particularly beneficial for patients with mixed-risk genotypes or complex clinical profiles, facilitating more precise irinotecan management [42]. Integrating *UGT1A1* testing within the broader framework of precision oncology represents a significant advance in personalized cancer treatment; as pharmacogenomic testing becomes more widely available, an increasing number of patients will benefit from individualized irinotecan dosing, leading to improved outcomes and reduced risks of severe toxicities. Although a guideline for UGT1A1 and irinotecan is currently not available, for patients carrying *UGT1A1*, guidelines recommend starting with a 30% reduced irinotecan dose, whereas there is a contraindication for an irinotecan dose of 240 mg/m^2^ or higher [39].

#### 2.4.3. Beyond Irinotecan: UGT1A1 in Other Cancer Treatments

While irinotecan is the most widely recognized chemotherapeutic agent influenced by *UGT1A1* polymorphisms, other oncology drugs also rely on UGT1A1-mediated metabolism, though to a lesser extent. These include certain hormonal therapies, such as estrogens and androgens, as well as some newer targeted therapies. However, the clinical significance of *UGT1A1* polymorphisms in the metabolism of these drugs is not as well established as it is for irinotecan [42,43,44].

The phase 3 ASCENT trial evaluated the safety of sacituzumab govitecan (SG) in patients with metastatic triple-negative breast cancer (mTNBC) who had relapsed or were refractory to at least two prior chemotherapy regimens. In an exploratory safety analysis, patients with the *UGT1A1* *28/*28 genotype experienced higher rates of grade ≥3 neutropenia, febrile neutropenia, anemia, and diarrhea compared to those with other genotypes [45]. Therefore, patients with the *UGT1A1* *28/*28 genotype treated with SG should be monitored closely.

**Figure 2:**
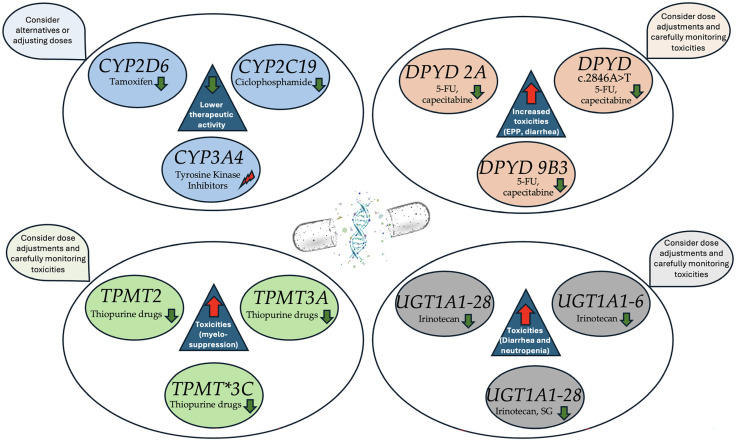
Key pharmacogenomic genes with clinical implications in solid tumors. Green arrows indicate a reduction in function, while red arrows indicate an increase. The beam represents dysfunction, which can imply either an increase or a decrease in enzyme function.

Figure 2 summarizes the main key pharmacogenomic genes and their clinical implications.

## 3. Ongoing Research and Future Directions

Current research in pharmacogenomics focuses on expanding *DPYD* and *UGT1A1* testing, exploring new genetic variants, refining treatment strategies, and reducing drug toxicity, with the goal of advancing personalized oncology and improving patient outcomes [26,46]. Routine genetic testing is not yet universally implemented despite its clear clinical benefits. That is why ongoing research aims to demonstrate its cost effectiveness and enhance testing accessibility [27].

In the case of DPYD, the development of rapid, cost-effective genetic assays could make widespread testing more feasible [26]. Although *DPYD 2A*, c.2846A>T, and other common variants have been well studied, research continues into additional polymorphisms that may contribute to DPYD deficiency. In particular, ethnic differences in DPYD variant prevalence highlight the need for population-specific testing protocols. For example, DPYD 2A is more common in European populations but less prevalent in Asian populations, which may require different testing approaches in diverse clinical settings [28,31].

Furthermore, research is underway to combine *DPYD* genotyping with phenotypic testing, such as measuring dihydrouracil (the metabolite of 5-FU) levels in plasma, to create a more comprehensive assessment of DPYD function [27]. This approach could improve the accuracy of identifying patients at risk of fluoropyrimidine toxicity, particularly in cases with novel or rare DPYD variants [28,29].

Researchers are also exploring the integration of *DPYD* testing with other genetic markers involved in fluoropyrimidine metabolism, such as thymidylate synthase (TYMS) and methylenetetrahydrofolate reductase (MTHFR) [43]. This multi-gene approach could provide a better understanding of individual drug response and toxicity risks, leading to even more precise and personalized treatment strategies [47]. The role of *DPYD* in pharmacogenomics is crucial for the safe and effective use of fluoropyrimidine-based chemotherapy. Genetic polymorphisms in DPYD can lead to significant variability in drug metabolism, with potentially life-threatening toxicities in individuals with deficient enzyme activity. Pre-treatment *DPYD* testing is now recommended in many clinical guidelines to identify at-risk patients and tailor chemotherapy regimens accordingly [47]. While the routine implementation of *DPYD* testing is not yet universal, ongoing research supports its importance in personalized cancer treatment, potentially improving patient outcomes and reducing treatment-related morbidity [31].

Ongoing research continues to investigate *UGT1A1* polymorphisms across various populations. For example, the UGT1A16 variant, which is more prevalent in Asian populations, significantly reduces enzyme activity, similar to UGT1A128 [37,38]. Understanding the prevalence and impact of these population-specific variants is critical for developing tailored pharmacogenomic guidelines across different ethnic groups [46]. Future studies will also focus on how UGT1A1 polymorphisms may impact the efficacy and safety of combination therapies, especially regimens that include irinotecan alongside other chemotherapeutic or targeted agents [38]. Understanding the interaction between multiple drugs and their metabolic pathways is crucial for optimizing combination treatments in oncology [40].

Although UGT1A1 is the most extensively studied isoform of the UGT family regarding drug metabolism, other isoforms, such as UGT1A4 and UGT1A9, may also contribute to drug metabolism and are being investigated for their potential impact on chemotherapy outcomes and toxicity [44]. As more pharmacogenomic data become available, pharmacogenomic testing for *UGT1A1* and other relevant genes is expected to be incorporated into broader gene panels that assess multiple pharmacokinetic and pharmacodynamic pathways. This holistic approach will allow oncologists to make more informed treatment decisions based on a patient’s genetic profile [38].

The emerging field of polygenic risk scores aims to improve the accuracy of personalized therapy by considering the cumulative effects of multiple genetic variants [48]. A growing field is the use of circulating tumor DNA (ctDNA) and circulating tumor cells (CTCs) for non-invasive pharmacogenomic testing. Liquid biopsies allow for real-time tumor evolution and drug-resistance monitoring, facilitating more dynamic treatment strategies [49]. Advanced computational models are being developed to understand the complex interactions between multiple genes and drugs. These models aim to predict not only drug efficacy but also potential adverse drug reactions based on an individual’s genetic profile [50].

The interplay between the immune system and cancer pharmacogenomics is a promising area of research. Identifying genetic markers that predict response to immunotherapies, such as immune checkpoint inhibitors, could revolutionize cancer treatment [38]. Advances in CRISPR-based gene editing are also offering valuable insights into gene–drug interactions and potential therapeutic targets in preclinical, providing valuable insights into gene–drug interactions and potential therapeutic targets [50].

## 4. Conclusions

Pharmacogenomics is a cornerstone of modern precision oncology, with the potential to transform cancer treatment by personalizing therapy based on genetic profiles. This review has highlighted the crucial roles of genetic markers such as *CYP2D6*, *DPYD*, and *UGT1A1* in influencing drug metabolism, efficacy, and toxicity. The clinical relevance of these biomarkers is evident in the management of breast, gastrointestinal, and lung cancers, amongst others, where pharmacogenomic testing can guide therapy and minimize the risk of severe adverse effects.

One of the key findings is the growing importance of *DPYD* and *UGT1A1* testing to improve drug safety, particularly in patients receiving fluoropyrimidines and irinotecan-based regimens. Novel multi-gene approaches, combining markers like *TYMS* and *MTHFR* with *DPYD* testing, offer a more comprehensive understanding of drug response and toxicity risks. Additionally, the development of liquid biopsies and advanced computational models holds promise for non-invasive pharmacogenomic testing and predictive analytics in oncology.

Looking ahead, the integration of pharmacogenomic data with broader genomic markers, polygenic risk scores, and liquid biopsies promises to further refine cancer treatments. The continued advancement of pharmacogenomics will be instrumental in achieving the overarching goal of oncology: delivering highly effective, minimally toxic, and personalized treatments that revolutionize cancer care and significantly enhance patient outcomes worldwide.

## Figures and Tables

**Table 1:** Clinical implications of cytochrome P450 (CYP) variants.

Gene (Enzyme)	Drug Affected	Cancer Type	Polymorphism	Clinical Implications	Recommendations
*CYP2D6*	Tamoxifen	Breast cancer	Poor metabolizers	Reduced conversion of tamoxifen to active metabolite (endoxifen), leading to decreased efficacy	Consider alternative therapy or increased dose of tamoxifen.
*CYP2C19*	Cyclophosphamide	Sarcoma, lymphoma, breast cancer	Poor metabolizers	Reduced activation of cyclophosphamide, potentially leading to lower therapeutic efficacy.	Adjust dosing or use alternative chemotherapeutic agents.
*CYP3A4*	Tyrosine kinase inhibitors (Imatinib, gefitinib)	GIST, lung cancer, sarcoma	Polymorphisms affecting activity	Altered drug metabolism (either enhanced clearance or toxicity due to poor metabolism).	Monitor drug levels closely; consider dose adjustments.

**Table 2:** Clinical implications of DPYD (Dihydropyrimidine dehydrogenase) variants.

Drug Affected	Polymorphism	Clinical Implications	Recommendations
5-Fluorouracil (5-FU), Capecitabine	*DPYD 2A* (c.1905+1G>A)	Reduced enzyme activity, leading to accumulation of 5-FU and severe toxicities (myelosuppression, GI toxicity, neurotoxicity).	Reduce starting dose or consider alternative therapies.
5-Fluorouracil (5-FU), Capecitabine	c.2846A>T	Moderate reduction in enzyme activity, associated with increased toxicity risk.	Adjust dose based on genotype or consider alternative treatments.
5-Fluorouracil (5-FU), Capecitabine	*DPYD 9B3* (c.1679T>G)	Partial deficiency in enzyme activity, resulting in increased risk of severe toxicity.	Dose reduction and careful monitoring for toxicity.

**Table 3:** Clinical implications of TPMT variants.

Drug Affected	Polymorphism	Clinical Implications	Recommendations
Thiopurine drugs	*TPMT2*, *TPMT3A*, TPMT*3C	Reduced TPMT enzymatic activity, leading to the accumulation of toxic thioguanine TGNs, increasing a the risk of developing severe myelosuppression	Dose reductions of up to 90% for poor metabolizers, and moderate dose reduction and careful monitoring for intermediate metabolizers

**Table 4:** Clinical implications of UGT1A1 (Uridine Diphosphate Glucuronosyltransferase 1A1) variants.

Drug Affected	Polymorphism	Clinical Implications	Recommendations
Irinotecan	*UGT1A1 28 (7/7)*	Reduced enzyme activity, leading to impaired SN-38 glucuronidation, increased risk of severe neutropenia, and diarrhea.	Reduce irinotecan dose by 30–50%; monitor closely for toxicity.
Irinotecan	*UGT1A1 28 (6/7)*	Intermediate enzyme activity, moderate risk of toxicity (neutropenia, diarrhea).	Consider moderate dose reduction or close monitoring.
Irinotecan	*UGT1A1 6 (Asian populations)*	Similar to UGT1A1 28, leading to reduced metabolism and increased toxicity.	Adjust dose based on genotype, particularly in Asian populations.
Sacituzumab govitecan	*UGT1A1 28*	Increased risk of neutropenia and diarrhea.	Monitor closely and consider dose reduction.
